# Pharmacological Effects and Toxicogenetic Impacts of Omeprazole: Genomic Instability and Cancer

**DOI:** 10.1155/2020/3457890

**Published:** 2020-03-28

**Authors:** Márcia Fernanda Correia Jardim Paz, Marcus Vinícius Oliveira Barros de Alencar, Rodrigo Maciel Paulino de Lima, André Luiz Pinho Sobral, Glauto Tuquarre Melo do Nascimento, Cristiane Amaral dos Reis, Maria do Perpetuo Socorro de Sousa Coêlho, Maria Luísa Lima Barreto do Nascimento, Antonio Luiz Gomes Júnior, Kátia da Conceição Machado, Ag-Anne Pereira Melo de Menezes, Rosália Maria Torres de Lima, José Williams Gomes de Oliveira Filho, Ana Carolina Soares Dias, Antonielly Campinho dos Reis, Ana Maria Oliveira Ferreira da Mata, Sônia Alves Machado, Carlos Dimas de Carvalho Sousa, Felipe Cavalcanti Carneiro da Silva, Muhammad Torequl Islam, João Marcelo de Castro e Sousa, Ana Amélia de Carvalho Melo Cavalcante

**Affiliations:** ^1^Postgraduate Program in Biotechnology (RENORBIO), Federal University of Piauí, Teresina, PI, Brazil; ^2^Laboratory of Genetic Toxicity, Postgraduate Program in Pharmaceutical Sciences, Federal University of Piauí, Teresina, PI, Brazil; ^3^University Centre UNINTA, Sobral, CE, Brazil; ^4^University Hospital, Teresina, PI, Brazil; ^5^Postgraduate Program in Pharmaceutical Science, Federal University of Piauí, Teresina, PI, Brazil; ^6^University Centre UNINOVAFAPI, Teresina, PI, Brazil; ^7^Laboratory of Genetics and Molecular Biology, Federal University of Maranhão, São Luís, MA, Brazil; ^8^Getúlio Vargas Hospital, Teresina, PI, Brazil; ^9^Department of Biological Sciences, Federal University of Piauí, Picos, PI, Brazil; ^10^Department for Management of Science and Technology Development, Ton Duc Thang University, Ho Chi Minh City 700000, Vietnam; ^11^Faculty of Pharmacy, Ton Duc Thang University, Ho Chi Minh City 700000, Vietnam; ^12^Department of Biochemistry and Pharmacology, Federal University of Piauí, Teresina, PI, Brazil

## Abstract

Omeprazole (OME) is commonly used to treat gastrointestinal disorders. However, long-term use of OME can increase the risk of gastric cancer. We aimed to characterize the pharmacological effects of OME and to correlate its adverse effects and toxicogenetic risks to the genomic instability mechanisms and cancer-based on database reports. Thus, a search (till Aug 2019) was made in the PubMed, Scopus, and ScienceDirect with relevant keywords. Based on the study objective, we included 80 clinical reports, forty-six *in vitro*, and 76 *in vivo* studies. While controversial, the findings suggest that long-term use of OME (5 to 40 mg/kg) can induce genomic instability. On the other hand, OME-mediated protective effects are well reported and related to proton pump blockade and anti-inflammatory activity through an increase in gastric flow, anti-inflammatory markers (COX-2 and interleukins) and antiapoptotic markers (caspases and BCL-2), glycoprotein expression, and neutrophil infiltration reduction. The reported adverse and toxic effects, especially in clinical studies, were atrophic gastritis, cobalamin deficiencies, homeostasis disorders, polyp development, hepatotoxicity, cytotoxicity, and genotoxicity. This study highlights that OME may induce genomic instability and increase the risk of certain types of cancer. Therefore, adequate precautions should be taken, especially in its long-term therapeutic strategies and self-medication practices.

## 1. Introduction

Cumulative reports suggest that a high prevalence of gastroesophageal diseases and drug-induced side effects may result in genomic instability (GI), leading to increased mutations and carcinogenesis [1–3]. Omeprazole (OME) therapy can alter the bacterial flora of the gastrointestinal tract, leading to malabsorption, enteric infections, and acute or chronic lesions in the stomach. This is due to the compensatory effect in response to decreased acid production, resulting in the destruction of the gastric glands and persistent hypergastrinemia, a denomination for atrophic gastritis [4].

Also, *Helicobacter pylori* infection and OME monotherapy can cause atrophic gastritis associated with an increased risk of mucosal dysplasia and gastric cancer [4]. Although these events may be derived by different mechanisms, a common theme is the involvement of reactive oxygen and nitrogen species (ROS/RNS) in the human stomach and oncoprotein production such as the cytotoxin-associated gene A (CagA) [5].

OME, especially for long-term use, may induce DNA damage [6, 7]. Genotoxicity assays have been shown that not only OME but all prazoles (e.g., esomeprazole, lansoprazole, pantoprazole, and rabeprazole) can induce chromosomal damages [8–11]. Upon understanding the overall fact, this review aimed to sketch a current scenario on the pharmacological effects and toxicogenetic risks of OME therapy in the context of genomic instability and cancer.

## 2. Methodological Strategies

We conducted a systematic review of published manuscripts to determine if exposure to OME during the treatment of gastric disorders increases the risk of genomic instability and cancer. The search criteria for this study includes publications in English using the keyword “Omeprazole,” which was then paired with “genomic instability,” “genotoxicity,” “cancer,” “gastritis,” “gastric ulcer,” and “gastric/stomach cancer,” in the PubMed, Scopus, and ScienceDirect databases. We excluded irrelevant reports that are not meeting inclusion criteria, duplicated publications, and data dealing with other prazoles than OME. The data obtained are listed in Table 1. Out of the 6349 articles, only 202 met our inclusion criteria (80 clinical reports, forty-six *in vitro*, and 76 *in vivo* studies). The selected articles were read in full.

## 3. Characterization of Scientific Reports

We have analyzed studies based on doses, side effects, drug interactions, pharmacological effects, and toxicogenetic risks (Table 2). The therapeutic use of OME is related to the treatment of duodenal ulcers, gastric ulcers, gastric cancer, and especially to gastroesophageal pathologies (42.4%) and others (26.0%). Regarding *in vitro* studies, the models are more related to other pathologies (90.0%), while for *in vivo*, most studies are associated with the simulating gastric pathologies. Few studies emphasize the use of antioxidants during OME therapy. Also, the therapeutic use of OME in clinical, *in vivo*, and *in vitro* studies varies between 10 and 40 mg/kg, 40 mg/kg, and 40 *μ*M to 25 mM, respectively.

Regarding mechanisms of OME therapeutic action, clinical studies emphasized mechanisms of proton pump inhibition (52.6%), acid and pH control (26%), and CYP219 and CP3AY enzyme inhibition, which are involved in the processes of OME metabolism. In a similar manner, *in vivo* studies are also correlated to proton pump inhibition (60%) and metabolizing enzymes (14.3%), although about 18% emphasized studies related to aryl hydrocarbon receptors (AhR). Around 27% of *in vitro* studies are about acid and pH control, and the same percentage for AhR and proton pump.

Clinical studies on toxicogenetic effects of OME are still limited (5.3%). However, about 89.5% of them point out to oxidative risks by ROS formation, which is also observed in *in vivo* studies. ROS-mediated cytotoxic effects on test systems were also seen in *in vitro* and *in vivo* studies (Table 3). In spite of the scarcity of toxicogenetic studies, the OME mechanisms of action were correlated to genotoxicity by applying bivariate correlation statistics, using the Spearman correlation factor of *r* = 0.433^∗^ and *p* < 0.044 in nonclinical studies and *r* = 0.577^∗^ and *p* < 0.005 in studies with cell cultures. At clinical doses, there were correlations with genomic instability (*r* = 0.300^∗^ and *p* < 0.032) and cytotoxicity (*r* = 0.532^∗∗^ and *p* < 0.001). In studies of drug interactions, toxicity was strongly correlated with the genomic instability (*r* = 1.000 and *p* < 0.001).

## 4. Anatomophysiological Characteristics of the Stomach

The stomach is divided into three portions: fundus, corpus, and antrum pylorus, where the processes of digestion, absorption, and protection take place. The lubrication and protection of the gastric mucosa are maintained by enzymatic activity, during digestive process that contribute to the maintenance of acidic pH by hydrogen ion secretion [12]. The pyloric and oxyntic glands act on the gastric mucosa. The former types are located in the antrum of the stomach and have the same cell types as the oxyntic glands, except the parietal cells that, when stimulated, release the gastrin, mainly responsible for the secretion of gastric acid [13]. The oxyntic glands, responsible for secreting hydrochloric acid (HCl), are located in the fundus and the corpus of the stomach. They consist of somatostatin-producing D cells; main cells, responsible for the secretion of pepsinogen; enterochromaffin-like cells responsible for secretion of histamine; parietal cells, which mainly secrete HCl and intrinsic factors; and mucosal cells, responsible for the secretion of mucus and bicarbonate ions [14]. The enterochromaffin cells are stimulated by gastrin or acetylcholine, releasing histamine, which binds to H2 receptors found in the parietal cells, stimulating the secretion of acid by the proton pump [15, 16].

Acetylcholine stimulates pepsinogen secretion by peptic cells, HCl by parietal cells, and mucus by the mucous cells [17]. The parietal cells, present in the gastric mucosa, when stimulated, are responsible for the secretion of HCl through the H^+^/K^+^ adenosine triphosphatase (H^+^/K^+^/ATPase–proton pump) from the canalicular membrane [18].

### 4.1. Alteration of Gastric Mucosa

#### 4.1.1. Inflammation

Gastritis is considered a superficial and inflammatory lesion that can also compromise the integrity of the stomach mucosa or duodenum and cause lesions in deeper layers, resulting in gastric ulcers [19] and stomach cancer [20]. The body has preepithelial defenses against gastric lesions and protective factors such as the production of bicarbonate and mucus, nitric oxide (NO), blood flow, prostaglandins, cell regulation, growth factors, nonprotein sulfhydryl groups (SHs), and antioxidant defenses.

It is noteworthy that the lesions may be caused by alterations in the balance between protection and aggression factors to the gastric mucosa [21]. Loss of mucosal protection, derived from the deficiency in mucus secretion and bicarbonate, favors the action of HCl [22]. Gastric secretion, pepsin, free radicals, bile reflux, and ischemic processes are aggressive factors to the tissue [23]. HCl and pepsin generate lesions in the gastric mucosa that destabilize the gastric barrier and cause acute inflammation [24].

Increased gastric HCl secretion is one of the most prominent lesion signals, and its reduction is the main strategy for preventing gastric lesions [25]. The unbalance between harmful (HCl and pepsin) and protector agents characterizes the acute inflammatory process [24, 26]. As a consequence of chronic gastritis and stomach inflammations, peptic ulcers and gastric cancer are the most frequent pathological alterations [20, 27], especially during *H. pylori* infections [27].

#### 4.1.2. Infection by Helicobacter pylori

*H*. *pylori* is described as a bacterium whose reservoir is the human stomach [28, 29]. It is a gram-negative bacillus, with flagella, adhesion factors, urease enzyme, cytosines, and proteases as virulence factors [30]. *H*. *pylori* produces toxic enzymes, as well as induce the release of gastrin, leading to an increase in gastric acid secretion and pH, stimulating somatostatin release [26] and hypergastrinemia. *H*. *pylori* also triggers a trophic effect and hyperplasia of the enterochromaffin and parietal cells [31].

Infection with *H*. *pylori* may cause gastritis, gastric and peptic ulcers, and even gastric cancer [32]. Gastric ulcer is considered one of the major public health consequences that occur due to many factors, especially the harmful activity of gastric acid and pepsin [33]. Peptic ulcer is characterized by acid peptic lesions in the digestive tract, which result in mucosa ruptures (reaching the submucosa) that are generally found in the proximal stomach or duodenum [34]. Gastric lesions associated with *H*. *pylori*, with exposure to acid or pepsin, are amplified and more aggressive [27]. *In vivo* studies indicate that the presence of *H*. *pylori* may lead to the maintenance of chronic inflammatory responses, as well as to other pathological disorders in the stomach mucosa [35].

The use of nonsteroidal anti-inflammatory drugs (NSAIDs), stress, smoking, excessive alcohol consumption, and the presence of *H*. *pylori* in the gastrointestinal tract may reach the deeper layers of the muscular wall of the gastric mucosa and cause gastric ulcers [19, 36].

## 5. Therapies for Gastric Lesions

Proton pump inhibitors (PPIs), such as OME, are frequently used in gastric therapies [37, 38]. Other PPIs, such as lansoprazole, rabeprazole, pantoprazole, esomeprazole, and dexlansoprazole, are also used to inhibit HCl secretion [39]. These drugs are considered efficient in suppressing gastric acidity [40]. PPIs present chiral sulfur in their chemical structure and are activators of the AhR and inducers of CYP1A metabolism genes in human hepatoma cells and primary human hepatocytes [41]. The product of these genes may influence the pharmacokinetics and pharmacodynamics of OME [42, 43].

PPIs activate and release sulfonamide or sulfenic acid, thus inhibit gastric acid secretion by covalently (irreversible) binding to the sulfhydryl group of cysteine in the extracellular domain of H^+^/K^+^-ATPase [44]. A reduction in gastric acid secretion results in a faster lesion healing, depending on the dose administered [45].

OME is a first-line drug for inhibiting gastric acid secretion in the treatment of gastroesophageal reflux disease (GERD), peptic ulcer, and *H*. *pylori* infection [46]. Its mechanisms of action occur from selective and covalent activation with H^+^/K^+^-ATPase, in particular of extracellular cysteine 813, leading to potent inhibition of gastric acid secretion and triggering changes in the stomach flora [38]. Another mechanism is by blocking the proton pump in the stomach parietal cells, activating the heat shock protein (HSP70), and the transforming growth factor beta (TGF-*β*) [47], with consequent relief of symptoms and lesion healing [48].

### 5.1. Therapeutic Effects of Omeprazole and Suggested Mechanisms

**Figure 1:**
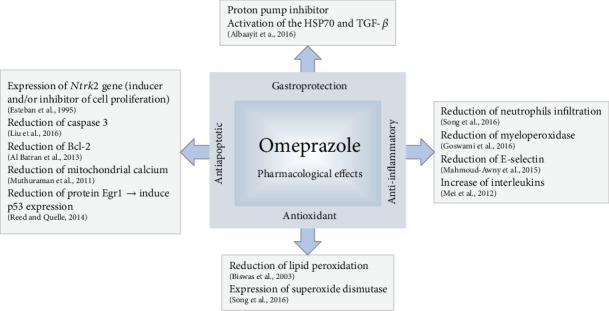
Pharmacological effects of omeprazole and suggested mechanisms of action.

Several therapeutic effects have been suggested for OME, such as gastroprotection [49], antioxidant [50], anti-inflammatory [51], antinecrotic [52], and antiapoptotic [53]. The mechanisms of action of these effects are presented in Figure 1.

#### 5.1.1. Gastroprotective Effect

OME gastroprotection is attributed to its ability to block the proton pump in the parietal cells of the stomach, activating the HSP70 and the TGF-*β* as mentioned above [47]. The expression of HSP70 mRNA was observed in the gastric tissue of rats pretreated with OME [54, 55]. This OME mechanism was reported for rats at doses that varied between 10 *μ*M and 400 mg/kg, as well as 200 *μ*M/ml in regular epithelial cell lines (MDCK) and mouse macrophage (RAW264.7) [55, 56].

In clinical studies, there is evidence of the OME gastroprotective function at doses of 5 to 40 mg/kg by mechanisms associated with interaction with H2 receptors [57], pH control [58], inhibition of CYP2C19 enzymes [42, 43], and histamine blockade [59]. OME in *in vivo* studies increased prostaglandins and sulfhydryl compounds [60], increased expression of BAX and caspases [61], and AhR and CYP1A expression [41]. These mechanisms of action may be related to other pathways that eventually cause genomic instability.  Table 4 shows the gastroprotective mechanisms of OME, including its possible association with apoptosis and necrosis, as well as the risk of genomic instability.

#### 5.1.2. Antioxidant and Anti-Inflammatory Effects

Several *in vivo* studies indicate antioxidant activities of OME, due to mechanisms associated with reduction of lipid peroxidation at doses of 2 to 5 mg/kg [50], 10 mg/kg [70], and 20 mg/kg [56]. Antioxidant activities of OME were also reported considering gastric lesions in animals [51] and *in vitro* studies in epithelioid MDCK, RAW264.7 [71], and U-87 cells [72].

OME also has antioxidant activities (*in vitro*), by blocking hydroxyl radical (^·^OH), preventing apoptosis and necrosis [73], inducing nicotinamide adenine dinucleic acid (NADPH) kinase oxidoreductase production [74], and increasing endogenous antioxidants [72]. *In vivo* and *in vitro* studies report inhibition of necrosis by activation of TNF-*α*, interleukin B [75], and proinflammatory cytokines [76]. OME at 20 mg/kg presented antioxidant activity through mechanisms associated with increased superoxide dismutase (SOD) enzyme production [77, 78], as well as glutathione peroxidase (GPx) and reduced glutathione (GSH) at 30 and 40 mg/kg [51, 79]. In ethanol-induced gastritis rats, OME modulated mucosal lesions through its antioxidant and anti-inflammatory activity [80]. However, clinical studies regarding OME antioxidant activities have not yet been reported.

There are reports of *in vivo* studies, in which OME had effects over increased blood flow of the gastric mucosa and expression of gastric glycoproteins [69] that is used as a precise and sensitive marker for the gastric mucosal status. Moreover, OME also plays a significant role as an antiacid, pepsin-resistant, and ulceration protector, which helps to protect the mucosal integrity [81] and reduces neutrophil infiltration [69].

Other mechanisms of action of OME, observed in animals and in cell cultures, relate to the anti-inflammatory marker cyclooxygenase (COX)-2 [77] and NSAIDs, which inhibit COX-1 and COX-2 that cause gastric ulcerogenic effects [82] and to the increase in the neurotrophic tyrosine kinase receptor type 2 (Ntrk2) [83]. OME also reduced TNF-*α* capacity (*in vivo*) [84]. OME can exert its anti-inflammatory effect through increasing the anti-inflammatory cytokines and autoimmune pathologies [85]. Proinflammatory cytokines, particularly TNF-*α* and nuclear factor kappa B (NF-*κ*B), are inducers of apoptosis [86]. NF-*κ*B pathway is correlated to gastric lesions in response to TNF-*α* and IL-1 signaling [77, 87]. TNF-*α* is involved in inflammatory induction, lesion, and carcinogenesis in several tissues, including the gastric mucosa [87].

In clinical studies, OME can also act in reducing the effects of proinflammatory markers, such as IL-1*β* [56, 72], monocyte chemoattractant protein-1 (MCP-1) [88], and IL-6 [87]. In cell cultures, esomeprazole has anti-inflammatory activity through mechanisms associated with suppression of proinflammatory proteins, including proteins of cell adhesion molecule 1, nitric oxide synthase, TNF-*α*, and interleukins (e.g., IL-1*β* and IL-6). The anti-inflammatory activity is associated with antioxidant activity by the induction of cytoprotective proteins induced by heme oxygenase-1 (HO-1), as well as by inhibition of fibroblast proliferation [89]. OME also has anti-inflammatory effects through reduction of E-selectin [56] and myeloperoxidase (MPO) [90], which can cause damage to proteins, lipids, and DNA through ROS formation [91]. In summary, other OME mechanisms of action as an antioxidant and/or anti-inflammatory and its protective effects and/or risk of genomic instability, as well as toxicity, are presented in Table 5.

#### 5.1.3. Antiapoptotic and Antinecrotic Effects

Apoptosis induction is one of the mechanisms for inducing acute gastric lesion [95]. OME presented antiapoptotic effects (*in vivo*) associated with reduction of caspase 3 expression [90], as well as reduction of BAX [54] and mitochondrial calcium [96]. Other studies indicate that OME has antiapoptotic activity in the gastric and intestinal tissues, showing reduction of lesions through antioxidant processes and anti-inflammatory activity through expression of *Ntrk2* gene (inductor/inhibitor of cell proliferation) [97] and reduction of protein Egr1, which influences the increase of protein p53 [98].

Mitochondrial permeability transition pore is associated with apoptosis due to free radical production, calcium accumulation [99], and increase in mitochondrial ATP, which is linked to the maintenance of cellular respiration [96] and reduction of mitochondrial cytochrome C [100]. OME in gastric lesion models reduced ulcerative lesions [50], the incidence of gastric hemorrhages [101], prevented visible lesions with edema, erosions, and necrosis in gastric endothelial cells [102, 103], and reduced vascular permeability [104]. OME exerted an antinecrotic effect in rats at 10 *μ*M and 60 mg/kg doses [52] by increasing the gastric mucosal barrier [60] and reducing the necrotic area induced by skin suspension in the animal model [105].

### 5.2. Adverse Effects of Omeprazole and Suggested Mechanisms

**Figure 2:**
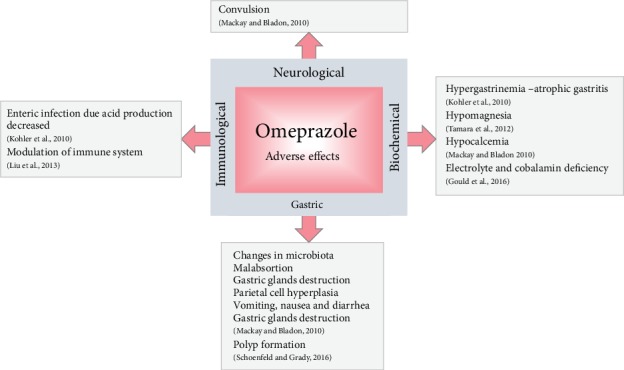
Adverse effects of omeprazole.

Figure 2 shows the adverse effects of OME. Evidences suggest that OME treatment can alter the gastrointestinal bacterial flora in response to decreased acid formation [106, 107] and increased gastrin production that causes hypergastrinemia. These events can result in gastric polyps, increased risk of bacterial infection, especially *H*. *pylori*, and gastric cancer as a consequence of the decreased somatostatin release from D cells [108].

Chronic therapies can induce electrolyte and cobalamin deficiency, interrupted bone homeostasis, hypergastrin, and acid secretion (rebound effect) in humans [109]. Atrophic gastritis is characterized by the destruction of the gastric glands and persistent hypergastrinemia [4].

Several studies have been reported that long-term OME use in the treatment of gastritis causes anomalies in the gastric mucosa, such as parietal cell hyperplasia, dilatation of canaliculi in the stomach fundus, corpus and antrum, and projection of cytoplasmic protrusions into the canaliculus lumen [110]. Common side effects observed in the literatures include headache, diarrhea, nausea, constipation, abdominal pain, pruritus, rebound acid hypersecretion, malabsorption, vitamin B_12_ deficiency, and hypotension.

Long-term use of PPIs is also associated with pathological alterations, such as protrusions of parietal cells, dilation of oxyntic glands [110], and development of fundic gland polyps, resulting from a trophic effect on parietal cells [111]. PPIs induce bone fractures [112], enteric infections [113], destruction of gastric glands that induce atrophic gastritis [114], being capable of compromising the immunological system [115], and increasing the risk of morbidity and mortality of patients [116].

Adverse side effects subsequent to long-term OME exposure include severe hypomagnesia [117] and hypocalcemia associated with vomiting, nausea, diarrhea, muscle cramps, and seizures are in relatively low frequency [118]. OME has effects against ulcerative damages induced in the gastric mucosa of rats and mice [56, 61], being able to block the proton pump in the parietal cells of the stomach [47] and activating HSP70 [119].

In summary, OME gastroprotective activities may induce various adverse effects reported in clinical studies such as diarrhea, nausea, constipation, immune deficiencies, fracture induction, vitamin B_12_ deficiency [120], allergies, respiratory infections, hypo- and hyperglycemia, and electrochemical changes [121]. It is important to emphasize that therapeutic clinical studies highlighted toxicity due to increased liver enzymes [122], while in *in vivo* and *in vitro* studies, toxicity has been reported by the oxidation of thiols, sulfonamides [123], caspase 3, and PARP cleavage [124]. Adverse effects of OME may be associated with apoptosis and tumor induction, immune changes, hyperplasia, inflammation, and polyp formation, which may imply in genomic instability (Table 6).

## 6. Toxicological Risks and Genomic Instability Induced by Omeprazole

Genetic variations induced by genomic instability are involved in the processes of initiation, progression, and resistance to therapy [132]. In the treatment of gastrointestinal disorders, drugs are intermittently or long-term used, and genotoxic risks must be assessed [6, 7]. Toxicogenetic assessment plays an important role in human health [133] and many drugs can be carcinogenenic due to the mechanisms associated with genotoxicity [134]. Thus, it is important in any drug therapy to evaluate the benefits against the risks, especially long-term drug treatment [6, 7].

**Figure 3:**
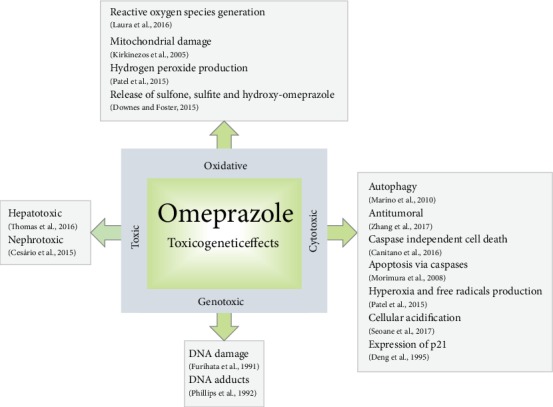
Toxicogenetic effects of omeprazole reported in clinical and nonclinical studies.

Studies in animals showed that some drugs induced genotoxicity through DNA damage, as well as micronuclei formation [10, 11, 135]. OME can induce DNA damage by mechanisms involved with oxidative damage, genotoxicity, and mutagenicity (Figure 3).

### 6.1. Oxidative Damage

Oxidative stress is an important parameter for chemical carcinogenesis [136]. ROS are continuously generated in cells through aerobic metabolism and exogenous sources, including drugs, pesticides, and other environmental factors [137]. This process occurs when the amount of substances responsible for oxidative damage exceeds the capacity of the endogenous antioxidant system [138]. As a consequence, there are alterations in the process of cell signaling, regulation, activation, apoptosis, and necrosis [139].

ROS can cause several types of damage in distinct biomolecules, including DNA, proteins, lipids, carbohydrates, and amino acids; cause ruptures, alterations in guanine and thymine bases, and translocations across the sister chromatids. These alterations can lead to inactivation of tumor-suppressing genes, such as *TP53* and *ATM*; or lead to increased protooncogenes gene expression [140]. ROS can also promote genomic instability and tumorigenesis through increased glucose metabolism and hypoxia adaptations and mutations, which contribute to the abnormal cell growth, angiogenesis, and apoptosis resistance [140].

There are reports that OME can amplify oxidative stress as a result of gastritis, damaging the gastric mucosa rather than accelerate its healing [4]. Excess ROS can result in inhibition of the gastric acid pump in parietal cells that leads to the release of sulfone, sulfite, and hydroxy-OME [141]. Effects of hyperoxia, inflammation, oxidative stress, and vascular lesions are amplified with OME administration of 25 mg/kg in rats along with the lung and alveolar vascular simplification promoted by AhR [142].

ROS are responsible for modifications in mitochondrial permeability [143], causing mutations and damage to the mitochondrial DNA and the respiratory chain [144, 145]. OME can impart cytotoxicity of hyperoxia and induce ROS in lung microvascular endothelial cells, by producing hydrogen peroxide rather than acute hyperoxic lesions (*in vitro*) [146].

### 6.2. Genotoxic Effects

Genomic instability caused by drugs can be associated with genotoxicity induction [6, 7, 134]. *In vivo* studies suggest that OME can promote DNA damage [147] through the formation of covalent adduct with DNA in experimental animals [148]. Assessment of genotoxicity of chemicals, including the identification of their mechanisms of action, is important to establish distinctions among carcinogens, especially in the pharmaceutical industry [134]. Drugs that are potentially inductors of genetic instability must have to be monitored before consumption [133].

CYP1A1-inducible chemicals, such as benzo [a] pyrene and 2,3,7,8-tetra-chlorodibenzo-dioxins, usually have adverse effects related to genomic instability (mutagenic, carcinogenic, and teratogenic). However, studies indicate that OME does not induce carcinogenesis, but it may amplify the effects of environmental carcinogens [148]. Nevertheless, studies on DNA damage and chromosomes are necessary and relevant [149], since *in silico* studies showed that OME can cause genotoxicity and mutagenicity through the formation of chromosomal aberrations and micronuclei [150].

At the molecular level, hypo- or achlorhydria triggers the formation of *N*-nitrosamines, which may induce DNA damage and provoke nuclear abnormalities, such as micronuclei, pyknosis, and karyorrhexis [41]. OME does not have a direct mutagenic effect [151], but DNA breaks are a result of oxidative stress [81] in events originated from elevated ROS levels, which cause oxidative damage to cell proteins, membrane lipids, and genetic materials (e.g., DNA, RNA) [152].

### 6.3. Toxicity of Omeprazole

To understand the mechanisms of drug toxicity, it is necessary to verify drug-drug interactions, the formation of reactive metabolites, and individual susceptibility by genetic polymorphisms in drug-metabolizing enzymes [153]. Gastric lesions are characterized by increased production of ^·^OH and protein oxidation, especially in gastric ulcers [73], which are related to lesion severity [78], producing highly toxic lipid derivatives that may modify cell function and even cell death [79].

OME induces hepatotoxicity in pregnant women, as observed by the reduction of aspartate aminotransferase (AST) and alanine aminotransferase (ALT) enzymes [122]. Hepatotoxic and nephrotoxic effects, thrombocytopenia, acute interstitial nephritis, anaphylactic reactions, gynecomastia, and impotence have been seen in the long-term OME use [154]. Several mechanisms are involved in drug hepatotoxicity; among those is the disassembly of actin fibrils that may result in cell lysis by changes in membrane transport pumps, as well as apoptosis by activation of the TNF-*α* [155]. Hepatic toxicity leads to hepatic lesions that disappear after discontinuation of the drug [156].

The PPIs can induce cytotoxicity through autophagic mechanisms, such as alterations in pH homeostasis [157]. OME presented cytotoxic effects in marine microalgae *Tetraselmis* sp. through hyperpolarization of cytoplasm and mitochondrial membranes, as well as by cell acidification and ROS generation [158]. Moreover, OME exerted toxic and cytotoxic effects in rabbit gastric gland cells that were attributed to oxidative processes [4].

More recent studies indicate that OME has antitumor activity against multiple myeloma as a single agent, and associated with chemotherapy, due to its cytotoxic activity as an apoptosis inductor, independently of caspases [159]. OME presented antitumor effects in association with chemotherapy in rectal cancer patients, including reducing the side effects of the treatment [160]. These effects were also observed in fibrosarcoma and colon cancer cells, suggesting that its use associated with anticancer drugs can be a promising therapy against malignant tumors [161].

Studies report that OME presents synergistic effects in rectal cancer chemotherapies [161]. Several mechanisms are proposed for the antitumor effect of Na^+^/K^+^ pump inhibitors [162], such as cell death stimulators *via* caspases, apoptosis inductors [163–166], an inhibitor of the V-ATPase activity, and turn tumors chemosensitive [167].

Antitumoral effects of OME were also described in studies with several cancer cell lines, including Heya8-MDR, SKOV3-TR, ES-2, and RMG-1; colon carcinoma cells (320WT and 320MUT) [168], neuroblastoma cells (SH-SY5Y), human microglia (THP), myeloma RPMI8226, U266, human gastric cancer (HGC-27), glioblastoma (U-87), human colon cancer (HCT-116 and HCA-7) cells, and *Jurkat T* lymphocytes at concentrations between 10 and 10^6^ *μ*M [169, 170].

In xenographic model of colon carcinoma and colon cells, antitumoral effects were also observed through increase expression of immediate early response gene X-1 (IEX-1), a stress-sensitive gene [171–173]. Other mechanisms have been suggested for the OME antitumor effect, such as reduction of Bcl-2 [174–176], Bcl-xL, and survivin [176], as well as reduction of other antiapoptotic proteins [177, 178].

In human gastric cancer cells (HGC-27) and polymorphonuclear neutrophils, OME exerted an antitumoral effect through increase in caspase 3 [123], apoptotic proteins [90], and cleavage of poly [ADP-ribose] polymerase 1 (PARP-1) [124, 169, 170]; OME was cytotoxic in colon cells through increased gastrin secretion and increasing expression of IEX-1. Moreover, OME showed antitumor effects in different carcinomas [172, 173] through mechanisms associated with reduction of Bcl-2 and Bcl-xL expression [176] and in chemoresistant cells (HeyA8-MDR, SKOV3-TR) in association with the anticancer drug paclitaxel [168].

In relation to genotoxicity in clinical studies, it has been reported for clastogenic effects and oxidative stress mechanisms [179] and hydroxylation induction and sulfoxidation in OME doses of 20 to 40 mg/kg [180]. Sulfonamide metabolites have also been reported as mechanisms for genotoxicity in *in vivo* studies [181]. Additionally, there are other mechanisms associated with DNA damage, including ornithine decarboxylase induction as a marker of cell proliferation [182], micronuclei induction [147], transcriptional changes [97], hyperplasia, hypertrophy, and other cellular alterations (Narimar et al., 2009).

OME antitumor effects in clinical studies are rare, but some have shown synergistic effects with antitumor drugs on modulating tumor acidity and apoptosis [160]. In *in vitro* studies, antitumor mechanisms have been related to expression of V-ATPase [168], inhibition of miR203-3p [183], and downregulation of metastatic CXCR4 proteins [184] and miRNAs [185]. In summary, other mechanisms indicative of genotoxicity, toxicity, and cytotoxicity of OME are shown in Table 7.

## 7. Carcinogenic Effects of Omeprazole

Severe pathological alterations on the stomach mucosa can lead to peptic ulcer and gastric cancer [20], especially due to complications with *H*. *pylori* infection and exposure to acid and pepsin [46, 107]. Gastric cancer is the 15^th^ leading cause of death by cancer, more frequent in men and mostly influenced by age, diet, and stomach diseases, including *H. pylori* infection [193].

Studies are still controversial, but *H*. *pylori* can be associated with gastric carcinoma by mechanisms related to increased ROS/RNS and oncoprotein formation [194]. Gastric pathologies are commonly related to increased levels of gastrin [195, 196]. Atrophic gastritis, resulting from monotherapy with OME in the context of *H*. *pylori* infection, has been associated with an increased risk of mucosa dysplasia and gastric cancer [114].

Carcinogenicity studies are preliminary to the approval and commercialization of pharmaceutical products, including cytogenetic *in vivo* and *in vitro* assays [197–199]. Brambilla and his colleagues report that, out of 535 medications, 279 showed positive results for carcinogenicity in animal tests. Thus, the indication of drugs should consider the risk/benefit in relation to the carcinogenicity and should prioritize new therapeutic intervention strategies [6–11, 200].

Studies have shown that after chronic gastritis, atrophy, intestinal metaplasia, and dysplasia, there are increased risks for gastric cancer [27, 57], especially with *H*. *pylori* infection [201]. Esomeprazole can induce acid suppression, leading to indigestion and amplifying risks of bacterial infections that generate atrophic gastritis [108, 202].

Long-term use of OME may relate to the cell proliferation and carcinoid tumors [203]. Menegasse et al. [204] concluded that proliferative changes of the oxyntic mucosa occur in individuals with chronic use of PPIs, with statistical significance in association with age and proliferative cell alterations [205]. Several studies reported that acid-suppressing drugs increase risks of polyp formation and/or gastric cancer due to nitrosamine production and hypergastrinemia. Decreased gastric acidity, due to gastric atrophy or to hypochloridria, may favor bacterial colonization, increasing the cancer risk [206]. OME (20 mg) induces gastric hyperplasia and polyps that increase with therapy and regress with discontinuation, independently of *H*. *pylori* infection [129].

Also, studies report that OME reacts with DNA and induces cancer in rodents [150]. OME, at high dose (30 mg/kg), was shown to induce carcinogenesis in the anterior stomach by influencing the levels of acid phosphatase (ACP) and *N*-acetyl-*β*-D-glucosaminidase (NAG) in the serum and spleen [207]. Other evidence showed that OME can induce hypergastrinemia and colorectal tumors [208].

PPIs are associated with *H*. *pylori*-induced chronic atrophic gastritis, metaplasia, and carcinoma [209]. Atrophic gastritis usually happens during monotherapies with OME, possibly resulting in dysplasia and gastric cancer [210].

In summary, other mechanisms of OME activity may also include carcinogenic risks due to its effects of hypergastrinemia and metaplasia [211]. Other mechanisms are also pointed out in *in vivo* studies such as ROS induction, oxidation of 8-DHD6 [212, 213], premalignant lesions [214], cell cycle alterations, genotoxicity, hyperplasia, and inhibition of liposomal hydrolases [215]. Several studies indicative of OME-mediated carcinogenicity are summarized in Table 8.

## 8. Conclusion

Studies on the mechanisms of action of OME are still controversial. As a gastroprotectant agent, it blocks proton pump, activates HSP70 proteins and TGF-*β*, exerts antioxidant activity, reduces lipid peroxidation, and activates expression of antioxidant defenses, without differentiation of doses and/or concentrations. Additionally, in *in vitro* and *in vivo* studies, anti-inflammatory effects of OME have been related to increased gastric flow, increased anti-inflammatory markers (COX-2, IL-10A, and IL-6), and antiapoptotic activity by reducing caspase 3, Bcl-2, mitochondrial calcium, and expression of *NTRK2* and *GGR1* genes. However, OME adverse effects, especially *in vivo*, such as changes in bacterial flora, enteric infections, gastric gland destruction, polyp formation, hypomagnesia, hypocalcemia, hyperplasia, intestinal metaplasia, electrolyte deficiency, and immunological component changes, may relate to the consequences of genomic instability. In summary, besides the gastroprotective effects, the adverse effects of OME may be due to its DNA damage capacity by inducing oxidative stress, apoptosis and necrosis, immunological alterations, cell proliferation, autophagy, and tumors.

## Figures and Tables

**Table 1:** Publications found in the databases.

Keywords (paired with OME)	Databases			Number of articles
	*PubMed*	*Scopus*	*ScienceDirect*	
*Genetic instability*	0	2	0	2
*Genotoxicity*	21	11	9	41
*Cancer*	94	1219	27	1340
*Gastritis*	605	2276	160	3041
*Gastric ulcer*	121	1311	87	1519
*Stomach/gastric cancer*	24	373	9	406
Total				6349

OME: omeprazole.

**Table 2:** Omeprazole studies published in scientific databases in relation to therapeutic use, mechanisms of action, dose/concentration, and interactions with vitamins.

Parameters	Clinical % (*n* = 80)	Nonclinical %	
		*In vitro* (*n* = 46)^#^	*In vivo* (*n* = 76)^##^
Analysis objects			
Dose	15.8	—	13.3
Adverse effects	10.5	9.1	13.3
Drug interactions	26.3	9.1	—
Mechanisms of pharmacological action	42.1^∗^	63.6^∗^	53.4^∗^
Toxicogenic risks	5.3	18.2	20.0

Therapeutic use			
Duodenal ulcer	15.8	—	26.7
Gastric ulcer	10.5	—	20
Gastroesophageal pathologies	42.4^∗^	9.1	20
Gastric cancer	5.3	—	13.3
Other pathologies	26.0	90.9	20.0

Mechanism of action			
Proton pump inhibition	52.6^∗^	27.3	60^∗^
Acid and pH control	26.3	27.2	7.4
CYP219 and CP3AY enzyme inhibition	10.5	—	14.3
Effect of gastric distension	5.3	—	—
Apoptosis and protein p53	5.3	—	—
Activators of the receptor (AhR)	—	18.2	18.3
Regulation ATPase in tumor cells	—	9.1	—
Inhibition of interleukin- (IL-) 8	—	9.1	—
Inhibition of absorption of Na^+^	—	18.2	—
Not reported	—	—	—

Dose/concentration			
10 mg/kg	5.3	—	—
20 mg/kg	66.7^∗^	—	6.7
30 mg/kg	8.7	—	6.7
40 mg/kg	19.3	—	20.2^∗^
20 mM	—	18.2	6.7
25 mM	—	18.2	6.7
40 mM/ml	—	—	20
100 mM	—	9.1	—
1 *μ*M	—	7.28	1.26
2 *μ*M	—	7.28	1.26
3 *μ*M	—	7.28	1.26
4 *μ*M	—	7.28	1.26
5 *μ*M	—	7.28	1.26
40 *μ*M	—	18.1	6.7
100 *μ*m/kg	—	—	10.0
200 *μ*m/kg	—	—	10.0

Interaction with vitamins			
Use of antioxidants	—	—	13.3
Without the use of antioxidants	100	100	86.7^∗^

^#^Concentration/ml. ^##^Dose/kg. CYP219 and CYP3AY (metabolizing enzymes). AhR: aryl hydrocarbon receptor; IL-8: interleukin 8. Chi-square test ^∗^*p* < 0.05.

**Table 3:** Characterization of omeprazole studies in relation to toxicogenetic effect, oxidative damage, and cytotoxicity.

Parameters	Clinical % (*n* = 80)	Nonclinical %	
		*In vitro* (*n* = 46)	*In vivo* (*n* = 76)
Toxicogenetic effect			
Mutagenicity	5.3	—	—
Interaction with catalase	—	9.1	—
Activation of AhR	—	9.1	13.3
Not reported	94.7^∗^	81.8^∗^	86.7^∗^
Oxidative damage			
Oxidation of thiols	10.4	18.2	20
Inhibition of cysteine interaction	—	9.1	—
Interaction and oxidation of cysteine residues	—	9.1	—
ROS induction	89.5^∗^	63.6^∗^	80^∗^
Cytotoxicity			
Oxidation of thiols	50.5	18.2	20
Oxidation of cysteine residues	49.5^∗^	18.2	-
ROS induction	—	45.4^∗^	80^∗^

AhR: aryl hydrocarbon receptors; ROS: reactive oxygen species. Chi-square test ^∗^*p* < 0.05.

**Table 4:** Gastroprotective effects of OME and mechanisms of action that may lead to protection and/or risk of genomic instability.

Dose/concentration	Study	Test system	Mechanisms of action	Prevention/risk of DNA damage	References
5-40 mg/kg	Clinical	Human (*n* = 94558)	H2 receptor antagonists and PPIs	Oxidative stress	[57]
20 and 40 mg/kg	Clinical	—	pH control	Not identified	[58]
20, 40, and 100 mg/kg	Clinical	Human (*n* = 12)	Inhibition of CYP2C19, pharmacokinetics, gastroprotection of microdoses	Oxidative stress	[42, 43]
10 mg/14 days	Clinical	Human (*n* = 32)	Gastroprotection	Not identified	[62]
20 mg/kg	Clinical	Human (*n* = 75)	Histamine blockage	Not identified	[59]
—	Clinical	Human (*n* = 17489)	Mechanisms involved in the gastric diseases	Oxidative stress	[63]
20 mg/kg	Clinical	Human (*n* = 70)	Pharmacokinetics-antiulceratives	Not identified	[64]
20 mg	Clinical	Human (*n* = 199)	Better action in patients with CYP 2 C1Q PM phenotype	Not identified	[65]
20 mg+amoxicillin 750 mg	Clinical	Human (*n* = 268)	Antacids, dose-dependent, CYP2C19 polymorphisms	Infection, oxidative stress	[66]
0.7, 1.4, and 4 mg/kg	*In vivo*	Horses	Pharmacokinetic and pharmacodynamic mechanisms	Not identified	[67]
15, 30, and 60 mg/kg	*In vivo*	Rats	Reduced necrotic damage, increased mucosal and gastric acid secretion reduction	Not identified	[52]
200 g/ml	*In vivo*	Rats	Increased prostaglandins synthesis and sulfhydryl compounds	Oxidative stress	[60]
40 mg/kg	*In vivo*	Rats	Inhibition of caspase 1, AC-YVAD-CMK, silencing of inflammasome NLRP3	Inhibition of apoptosis	[61]
40 mg/kg	*In vivo*	C57BL1 mice (*n* = 6)	Upregulation of BAX and caspase 3 → increased cell necrosis	Induction of apoptosis and necrosis	[61]
20 mg/kg	*In vivo*	Rats	Gastric protection, inhibition of H^+^/K^+^-ATPase system	Not identified	[68]
15 mg/kg	*In vivo*	Rats	Decreases blood flow, increased glycoproteins, prostaglandins, necrosis factor (TNF-*α*)	Not identified	[69]
1-100 *μ*M	*In vitro*	Human hepatocyte cell line	Activation of AhR and induction of CYP1A	Catalytic activities	[41]

PPIs: proton pump inhibitors; TNF-*α*: tumor necrosis factor-alpha.

**Table 5:** Antioxidant and/or anti-inflammatory activities of OME and its protective effects and/or risk of genomic instability.

Activities	Dose/concentration	Study	Test systems	Mechanism of action	Preventive approach	References
Antioxidant	2, 10, and 20 mg/kg	*In vivo*	Rats	Induction of CYP1A1, antihyperoxia	Prevention of oxidative damage	[92]
Antioxidant	10.0 *μ*M	*In vitro*: cell culture	Human lung fetal cells	Upregulation of NADPH kinase oxidoreductase-1 *via* Nrf-2 expression not dependent on Nrf-2	Prevention of oxidative damage	[74]
Antioxidant	2 and 5 mg/kg (dose-dependent)	*In vivo*	Rats	^·^OH scavenging capacity, prevention of apoptosis by nuclear fragmentation	Prevention of oxidative damage and apoptosis	[73]
Antioxidant/anti-inflammatory	8.49 g/ml	*In vivo*	Rats	Reduction of hemorrhages and inflammation, preserving the endoplasmic reticulum	Protection of oxidative stress	[80]
Antioxidant Antineuropathic	50 mg/kg	*In vivo*	Rats	Inhibits NF-*κ*B, releases cytokines, protects cranial cruciate ligament (CCL) damage induction, reduces oxidative stress, increases several internal antioxidants	Protection of oxidative damages	[72]
Antitoxicity	5 *μ*g/ml	*In vitro*	Tumor cells	Cytochrome P450 metabolism (CYP450), CYP2C19, CYP3A4, C4P2CY	Toxicity prevention	[93]
Anti-inflammatory	300 *μ*M	*In vivo*	Mice	Inhibition of TNF-*α* and interleukin	Antiapoptosis prevention of oxidative stress	[75]
Anti-inflammatory	Not reported	*In vivo*	Microglia	Inhibition of proinflammatory cytokines	Prevention of oxidative damage	[76]
Anti-inflammatory	0.5, 1.5, and 10 *μ*g/ml	*In vitro*	MRC-5 cells	Antibacterial effect	Protection from bacterial infection	[94]

**Table 6:** Mechanisms of adverse effects of omeprazole, which may be associated with prevention and/or risk of genomic instability.

Dose/concentration	Study	Study model	Mechanisms of action	Prevention/risk for genetic material	References
10 and 20 mg/kg	Clinical	—	Proton pump and histamine receptors, hyperplasia, gastric atrophy, carcinoid tumors	Apoptosis, tumor induction	[125]
—	Clinical	Human (*n* = 113)	Characterization of *H. pylori* associated with gastritis, therapeutic complications	Not reported	[126]
—	Clinical	Meta-analysis review	Hypomagnesemia	Not reported	[42, 43]
5, 10, 20, and 40 mg/kg	Clinical	Human (*n* = 764)	Adverse effects: diarrhea, nausea, constipation, immune deficiencies	Immunological changes	[127]
5 and 40 mg/kg	Clinical	Human (*n* = 170) (review)	Induction of fractures, vitamin B_12_ deficiency, and diarrhea	Apoptosis	[120]
20 mg/kg	Clinical	Patients with gastric disorders, case studies	Induction of allergies, respiratory infection, hepatotoxicity, electrochemical changes, hypo- and hyperglycemia, diarrhea	Apoptosis	[121]
20 and 40 mg/kg	Clinical	Case study	Deficiency of vitamin B_12_, anemia	Not identified	[128]
20 mg/kg	Clinical	Case study	Induction of gastroesophageal reflux	Metastases, hyperplasias, polyp	[129]
—	Several	Several	Intestinal nephritis, hepatitis, polyps, metaplasia, pneumonia	Cancer	[130]
—	Clinical	Human (*n* = 298)	Adverse effects on cysts and polyps	Lung cancer and pancreatic cancer	[131]
0.83–1.6 mg/kg	*In vivo*	Cats	Heartburn, hypergastrinemia, hypersecretions	Oxidative stress	[109]

**Table 7:** Mechanisms indicative of genotoxicity, toxicity, and cytotoxicity of OME and their implications for prevention and/or risk of genomic instability.

Activities	Dose/concentration	Study	Study model	Mechanism of action	Prevention/risk for genetic material	References
Genotoxicity	20 and 40 mg/kg	Clinical	Endoscopy biopsy	DNA damage, clastogenic effects, oxidative stress	Genomic instability, genetic risks	[179]
Genotoxicity	20 and 600 mg	Clinical	Human (*n* = 57)	Interaction between genetic variations, CYP2C19 hydroxylation, and sulfoxidation	Oxidative stress	[180]
Genotoxicity	20 mg/kg	Clinical	Human (*n* = 33)	Cytogenetic change: micronuclei formation	Genomic instability	[186]
Genotoxicity	20 mg/kg	*In vivo*	Rats	Cytogenetic alterations, breaks of sister chromatids, micronucleus formation, chromosomal alterations	Genetic instability, cytogenetic damage	[186]
Genotoxicity	—	*In vivo*	Rodents	Sulfonamide metabolites	Reactivity with DNA	[150]
Genotoxicity	1-100 *μ*M	*In vivo*	Rats	Activates sulfonamide groups, inhibition of DNA synthesis	DNA damage	[181]
Genotoxicity	30 and 100 mg/kg (p.o.)	*In vivo*	Rats	DNA synthesis, oxytocin decarboxylase induction	Cell proliferation	[182]
Genotoxicity	30 mg/kg	*In vivo*	Rats	Micronuclei formation, cellular alteration, cell proliferation	Chromosomal instability, genomic instability	[147]
Genotoxicity	10 and 100 mg/kg	*In vivo*	Rats	Cell proliferation and replication	Genomic instability	[187]
Genotoxicity	—	*In vivo*	Rats	Transcriptional changes in the gastric mucosa	Changes in inflammatory regulation genes and immune response	[97]
Genotoxicity	20 ml/kg	*In vitro*	Rats	Hyperplasia	Genomic instability	[188]
Toxicity	40 mg/kg	Clinical	Case study	Increased ALT and AST levels	Induction of apoptosis	[122]
Toxicity	—	Clinical	Human	Inflammatory, CYP2CI9 enzyme variation, acute nephritis	Genomic instability	[189]
Toxicity	30 and 60 mg/kg	Clinical	—	Microsomal hepatic inhibition, oxidase function, blocking of H^+^/K^+^-ATPase system	Oxidative damages	[190]
Toxicity	—	Clinical	Human (*n* = 2,634)	Interaction between anti-inflammatory and proton pump inhibitors	Apoptosis	[191]
Toxicity	40 mg/kg	Clinical	Human	Neutropenia	Nontoxic effect	[191]
Toxicity	100 *μ*M	*In vivo*	Rats	Oxidation and toxicity, thiol oxidation, conversion of OME to thiolytic sulfonamides, binding to cysteine residues of H^+^/K^+^-ATPase system	Oxidative damages	[192]
Toxicity	0.0001 and 50 mM	*In vitro*	Polymorphonuclear neutrophils	Apoptosis, sulfhydryl groups	Apoptosis	[4]
Toxicity	0.0001 mM	*In vitro*	Jurkat cells, lymphomas	Cleavage caspase 3 and PARP	Apoptosis	[123]
Antitumoral neoadjuvant	20 and 40 mg/kg (i.v.)	Clinical	Human (*n* = 127)	Modulation of tumor acidity, apoptotic cell death	Inhibition of cell proliferation	[124]
Antitumoral	80 mg/kg	Clinical	Human (*n* = 94)	Synergistic effects with antineoplastic drug	Apoptosis	[160]
Antitumoral	50, 100, and 200 *μ*M	*In vitro*	Human melanoma cells	Cytotoxic effect	Apoptosis	[87]
Antitumoral	10-40 mg/kg	*In vitro*, *in vivo*	Ovary cancer (*n* = 44) patients	Expression of V-ATPase, inhibition of V-ATPase mRNA protein	Apoptosis and cytotoxicity	[159]
Antitumoral	100 *μ*g/ml	*In vitro*	CP-A (ATCC CRL-4027) CP-B (ATCC-CRL4028) cells	Inhibits cell cycle growth (arrest cell cycle at G0/G1) by inhibiting miR203a-3p	Induction of apoptosis	[168]
Antitumoral	200 and 300 *μ*M	*In vitro*	Breast cancer (MCF, SKBR₃ MDA–MB-468) cell lines	Decreases MDA-MB, decreases expression of prometastatic proteins and the expression of C-X-C chemokine receptor 4 (CXCR4)	Prevention of metastasis and inhibition of cell proliferation	[183]
Antitumoral	10 mg/kg	*In vivo*	Rats	Decreases NO levels, decreases the expression of TNF-*α* and B catechins	Apoptosis	[184]
Antitumoral	10 and 30 mg/kg	*In vitro*	HeLa cervical cancer line	Expression of ATPase *via* SiRNA	Cell proliferation	[70]
Antitumoral	50 and 200 *μ*g/ml	*In vitro*	Pancreatic cancer cell lines	Interaction with ATPase function regulators, modulation of liposomal transport	Apoptosis	[22]
Antitumoral	100, 200, and 300 *μ*M/l	*In vitro*	Esophageal adenocarcinoma (KYSE410)	Control intra and extracellular pH, expression of miRNAs	Antiproliferative effect	[165]
Antitumoral	160 *μ*M	*In vitro*	Melanoma cells	Acidification and alkalinization of tumors, NADPH oxidase dysfunction	Autophagy, oxidative stress	[185]

**Table 8:** Mechanisms of action of omeprazole implicated in genomic instability, which are associated with cancer risks.

Dose/concentration	Study	Study model	Mechanism of action	Prevention/risk for genetic material	References
100 mg/kg	*In vivo*	Rats	Hypergastrinemia and pancreatic metaplasia	Genomic instability	[216]
20 mg/kg	Clinical	Case study	Hyperplasia, gastric carcinoma, hypoacidity	Cell proliferation	[211]
Not reported	Clinical	Human (*n* = 230) patients with *H. pylori*	Metaplasias, gastric atrophy	Gastric cancer	[217]
276 mg/kg	*In vivo*	Rats	Induction of ROS. 8-0Hd6	Apoptosis Tumors	[212, 213]
—	Several	Several	Premalignant lesions	Genetic alterations	[214]
30 mg/kg	*In vivo*	Rats	Inhibition of lysosomal hydrolase activity decreases P21 and mammalian target of rapamycin (mTOR) in the stomach	Changes in apoptosis and cell cycle	[215]
—	*In silico*	Artificial system	Formation of metabolites	Genomic instability	[218]
